# The care delivery experience of hospitalized patients with complex chronic disease

**DOI:** 10.1111/hex.12085

**Published:** 2013-05-27

**Authors:** Kerry Kuluski, Sylvia N. Hoang, Alexis K. Schaink, Celeste Alvaro, Renee F. Lyons, Roy Tobias, Cécile M. Bensimon

**Affiliations:** ^1^ Bridgepoint Collaboratory for Research and InnovationBridgepoint HealthTorontoONCanada; ^2^ Institute of Health Policy, Management and EvaluationUniversity of TorontoTorontoONCanada; ^3^ Evidence Development and StandardsHealth Quality OntarioTorontoOntarioCanada; ^4^ Department of Architectural ScienceFaculty of Engineering and Architectural ScienceRyerson UniversityTorontoONCanada; ^5^ Dalla Lana School of Public HealthUniversity of TorontoTorontoONCanada

**Keywords:** care needs, complex chronic disease, health care delivery, hospital, patient experience, qualitative

## Abstract

**Objective:**

This study investigated what is important in care delivery from the perspective of hospital inpatients with complex chronic disease, a currently understudied population.

**Participants and Setting:**

One‐on‐one semi‐structured interviews were conducted with inpatients at a continuing care/rehabilitation hospital (*n* = 116) in Canada between February and July 2011.

**Design:**

The study design was mixed methods and reports on patient characteristics and care delivery experiences. Basic descriptive statistics were run using SPSS version 17, and thematic analysis on the transcripts was conducted using NVivo9 software.

**Results:**

Patients had an average of 5 morbidities and several illness symptoms including activity of daily living impairments, physical pain and emotional disturbance. Three broad themes (each with one or more subthemes) were generated from the data representing important components of care delivery: components of the care plan (a comprehensive assessment, supported transitions and a bio‐psycho‐social care package); care capacity and quality (optimal staff to patient ratios, quicker response times, better patient–provider communication and consistency between providers) and the patient–provider relationships (characterized by respect and dignity).

**Conclusions:**

As health systems throughout the industrialized world move to sustain health budgets while optimizing quality of care, it is critical to better understand this population, so that appropriate metrics, services and policies can be developed. The study has generated a body of evidence on the important components of care delivery from the perspectives of a diverse group of chronically ill individuals who have spent a considerable amount of time in the health‐care system. Moving forward, exploration around the appropriate funding models and skill mix is needed to move the evidence into changed practice.

## Introduction

People think it's an easy solution. Medicate someone to the point of not feeling and the problem is solved. Many people could do with talk therapy, and it should be part of a complex, comprehensive rehab program. When you're in a car accident and your body gets broken, you become broken as well. (Hospitalized patient with complex chronic disease)

This quote articulates a key finding that emerged from a large collection of semi‐structured hospital‐based patient interviews. It challenges the historical definition of health as merely the absence of disease. Health encapsulates physical, mental, emotional and social health^1^ and an ability to adapt to ones environment.^2^

A growing number of individuals are living longer with one or more chronic diseases.^3^ A proportion of this population can be classified as having *complex chronic disease* (*CCD*) due to a combination of functional,^4^, ^5^ social 6 and/or mental health challenges.^7^ Individuals with CCD tend to require care from multiple health and social service providers (across many organizations) as well as family members who require the tools to respond to their fluctuating needs. Needless to say, acute focused health‐care systems are seldom equipped to respond to the range of needs of the CCD population.^8^ The needs of individuals with CCD tends to carry a huge financial burden due to the health services required,^9^ complicated treatment and medication regimens,^6^ lost productivity 10 and strain on social and familial networks.^11^

Although individuals with CCD represent one faction of the heaviest users of health system resources,^12^ very little is known about their actual care experiences.^13^ In fact, this population tends to be excluded from research, including clinical trials, due to the presence of multimorbidity and other complications.^14^ A 2012 paper entitled ‘*Multiple Conditions: Exploring literature from the consumer perspective’* written by Walker^15^ noted no articles that focused on the experience of individuals with multiple conditions. However, our team did uncover some literature that speaks to the experience of these patients. These studies noted that compared to the general population, individuals with multimorbidities are more likely to experience cost barriers, poorly coordinated care and inadequate communication from care providers.^16^, ^17^ Key perceived needs of these individuals include better access to providers through multiple modalities such as in‐person, internet or telephone^13^; personalized self‐management support^16^, ^18^; clearly communicated and individualized care plans^13^; system navigation and coordination to manage multiple providers and services^13^, ^16^, ^17^; continuity of care with a consistent care team^13^; and seamless discharges^16^ with linkages to needed homecare and specialist support.^19^ Assistance in dealing with conflicting and complex treatment and medication regimes^18^ and less out‐of‐pocket expenses^19^ are also cited as key needs. Greater availability of health human resources and transportation to get to needed services^18^ has been reported among individuals with complex needs in rural, under‐serviced areas.

These studies provide insight into patient experience and complexity (through multimorbidity) but are limited to community‐dwelling samples. An emerging programme of research led by Dr. Alison Kitson at the University of Adelaide on the fundamentals of nursing care confirms that there is a gap in our understanding of what is important in care for patients undergoing care in complex environments.^20^

The purpose of this study was to fill this gap by identifying what hospitalized patients report as being important elements in their experience of care and what would make it better. All individuals in this setting met our operational definition of CCD: one or more chronic health problems and on‐going impairments that require health services.

## Methods

### Study setting

The study took place at a Canadian hospital that provides continuing care for individuals following an acute care hospital stay. The hospital provides care for two streams of patients: complex rehabilitation and complex continuing care. Patients in the complex rehabilitation programme are typically recovering from hip and joint replacement, multiple fractures, functional loss from stroke or acquired brain injury. Complex continuing care patients include those with advanced diabetes, HIV/AIDS, progressive neurological and degenerative conditions, severe stroke or advanced dementia.

### Design and sampling

The study was a cross‐sectional design including a semi‐structured survey that was conducted with a convenience sample of hospital inpatients. Research assistants worked closely with hospital unit care managers who identified patients who were eligible to participate in the research. Given the nature of the population in the hospital of study, all were eligible to participate with the exception of those who could not provide informed consent including individuals with severe cognitive impairments. Approximately 3% of the hospital population met such criteria at the time of the study. Each interview was conducted by a trained interviewer, was audio‐recorded and transcribed verbatim. Due to the length of the interview, completion of data collection, at times, spanned multiple visits. The results reported in this article were embedded in a large hospital quality improvement study/needs assessment; thus, the sample size is much larger than other studies that report qualitative findings.

### Data collection tool

The data collection tool was a self‐designed survey and consisted of a mix of standardized scales, closed‐ and open‐ended questions. The design of the tool was based on a conceptual framework developed by the research team. The framework evolved from a scoping review of the literature and meetings with experts on the meaning of complexity in chronic disease.^21^ Detailed findings on the scoping review and the conceptual framework were published in a separate paper.^21^

Given the breadth of data collected, this article focuses exclusively on the responses to the parts of the survey that garnered information on what patients felt was important in care delivery. Three questions on the survey tapped into the care delivery experience: (i) ‘Can you tell me what might help you with your health conditions?’ (ii) ‘Based on your experience, what information would be helpful to patients receiving care at (hospital name)?’ (iii) ‘What do you think health‐care providers should know about caring for patients like you?’

### Data analysis

There were two parts to the analysis. First, frequency counts were calculated using SPSS version 17.0 on socio‐demographics (sex, age, education, marital status and ethnicity), morbidity count, number of illness symptoms, perception of health status and length of stay at the time of interview. Second, the sections of the interviews related to the important aspects of care delivery were extracted by the lead author (KK). When the patients commented on the important aspects of care delivery, it was not always in direct response to the allotted survey questions; thus, any additional text, throughout the interview, that pertained to the theme ‘what is important in care delivery’ were included in the analysis.

The extracted sections of the interview were then coded by two independent reviewers using both thematic and axial coding techniques.^22^ After reading the relevant text from the transcripts several times, both reviewers classified the text into themes. Themes that were not deemed relevant to the focus of the paper were excluded. For example, one such theme focused on the building design and context of care. The intent of the research was to understand the care experience of patients with CCD, not the building/context in which care took place. Although an important element, it was the intention of the team to focus on themes related to direct provision of care and interactions with health professionals. The data that pertained to the built environment will be presented in a separate paper.

The themes were then compared and negotiated until consensus was reached. Similar themes were combined, renamed and agreed upon by the two reviewers. Data were coded using NVivo software version 9.

## Results

One hundred and sixteen patients participated in interviews at the hospital of study between February and July 2011. One hundred and fifty‐eight patients were approached, and 116 consented to participate garnering a response rate of 73%. Of the 158, three patients were discharged before the interview took place, while 36 others refused due to fatigue or expressed lack of interest. 110/116 patients commented on the aspects of care delivery that were important to them and were captured in the qualitative analysis.

As noted in Table 1, the study sample included a mix of complex continuing care (68%) and rehabilitation patients (32%); males (42%) and females (58%); across young (12%), midlife (47%) and older age (41%) groups; and were largely Caucasian (73%). Patients had an average of five health conditions combined with an average of six illness symptoms from a 10‐item checklist: pain, weakness, emotional upset, illness related symptoms, physical mobility problems, difficulty with activities of daily living, difficulty with use of equipment or devices, difficulty paying attention, sensory challenges and difficulty carrying on a conversation. Almost half (49%) perceived that their health had declined in the last year. At the time of interview, participants had been in hospital for an average of 3 months.

**Table 1:** Patient characteristics

Variable	*N*	%
Sex	116	
Male	49	42
Female	67	58
Age category	111	
Young (up to 44)	13	12
Midlife (45–64)	52	47
Older (65+)	46	41
Education	109	
High school of less	47	43
More than high school	62	57
Marital status	112	
Has a partner (married or living common law)	30	27
Does not have a partner (unmarried, divorced, widowed, single)	82	73
Ethnicity	111	
** **Caucasian	89	73
Other	22	27
Morbidities	M = 5	
	Med = 5	
	Mode = 5	
	SD = 2.113	
Number of illness symptoms (checklist of 10 items)^a^	97	
	M = 6	
	Med = 6	
	Mode = 6	
	SD = 2.335	
Perception of health status (since one year ago)	112	
Worse	55	49
Same	30	27
Better	27	24
Length of stay at the time of interview (days)	M = 162.27^b^	
	Med = 66.5	
	Mode = 59	
	SD = 317.18	

a pain, weakness, emotional upset, illness‐related symptoms, physical mobility problems, activity of daily living impairments, difficulty with use of equipment or devices, difficulty paying attention, sensory challenges and difficulty carrying on a conversation.

b Approximately 3 months.

Three overarching themes (each with one or more sub‐themes) were gleaned from the data and represent areas that respondents felt need to be addressed to improve the care delivery experience: (i) components of the care plan, (ii) care capacity and quality, and (iii) the patient–provider relationship. Although it was not the intent of the study to look at thematic categories by socio‐demographic factors and disease complexity, we do note, however, that the three broad themes encapsulated the views of individuals across sexes, age categories, education levels, marital statuses, ethnicities, CCC/Rehab streams and perceived health severity. There do appear to be some emerging differences in the subthemes, particularly for the young–midlife population, which we discuss throughout. The lead author (KK) examined the socio‐demographic mix of individuals represented within each of the three broad themes by selecting out each transcript from each of the themes and linking it back to the socio‐demographic characteristics collected in the interview. The quotations provided in the results below were purposely chosen to demonstrate both thematic content as well as the variation of patients within our sample. The themes with illustrative examples are also outlined in Table 2. Transcription conventions include *[text within square brackets]* to provide clarification as well as *[…]* to illustrate text that has been deliberately omitted (due to redundancy).

**Table 2:** Themes and Findings

Theme	Subtheme	Examples
Components of the care plan	Targeted/comprehensive assessment	Integrate information collected to avoid overlap
		Include the history of the patient with help from family caregiver (if available)
	Supported transitions	Expectation management upon admission and discharge from hospital
		Coaching around readiness for next point of care and assistance with linking to appropriate resources
	Bio‐psycho‐social care package	Social and mental health support, not just physical rehabilitation
		Extra opportunities to do engage in physical rehabilitation
		Goal coaching and expectation
		management embedded in the care plan
		Care navigator
Care capacity and quality	Optimal staff to patient ratio	Quicker response times
	Better provider communication	Messages not reaching the provider leading to delays in response times
		Providers who speak and understand the English
	Consistency between care providers	Some providers go the extra mile while others seem to do the bare minimum
The patient–provider relationship	Respect and dignity	Looking beyond the ailment to the person
		Patient as an active contributor to the care experience
		Attention to patient dignity (particularly around sensitive types of care such as bathing)

### Components of the care plan

#### Comprehensive assessment

Lack of integration and appropriateness of data collected during assessments can lead to confusion and dissatisfaction among patients. Many patients highlighted these issues and commented on the length and redundancy of the initial assessment process once they entered the hospital. A 43‐year‐old female patient who was suffering from a long‐term degenerative condition described her initial days in hospital:

The first, second, third day, you are here, you'll probably meet eight different people. And you should see the overlapping questions. Why can't you correlate everything you've got to ask to one person?

(Transcript #11)

Likewise, a 59‐year‐old female patient who had a combination of diabetes, joint problems and wounds felt overwhelmed at the beginning of her hospital stay.

When a new patient comes in here, they do everything so fast that it's hard to comprehend all the information that you're getting. Like within a day or 2 days, I had spoken to everybody. And I didn't know who the recreational thing was and who was this and who was that.

(Transcript #12)

Others felt that important elements of the patient assessment were missing including key questions such as ‘*What is your objective?’ ‘How can we help you?’* as well as the history of the patient. A caregiver of an 87‐year‐old man suffering from multiple morbidities and joint problems discussed the importance of garnering a true understanding of the history of the patient.

Caregiver: ‘Well, they should know the whole history because if you don't know the whole medical history, you won't really know how to help a person […] but I mean I've been with [my partner/husband] all the time. I know exactly what he needs and what he can and what he cannot do’.

(Transcript #68)

#### Supported transitions

As patients moved into and out of the hospital, they expressed the need for additional information, including what to expect during their time in hospital and at their next point of care. Patients noted that such information would mitigate feelings of uncertainty and stress during the care process. A 35‐year‐old female patient who was recovering from a brain tumour spoke about her first few days in hospital.

I didn't know what to expect at all. I knew I was coming. I knew I could get some speech assistance. I didn't know I'd get physio. I didn't know I'd get occupational [therapy]. I didn't know much as far as even their scheduling. I didn't know how big the building was, who they had here, like what type of individuals.

(Transcript #35)

Patients made suggestions on how to ease the transitions experience including the use of ‘peer supports’ (having current hospital patients help new patients ease into their new, unfamiliar environment).

Among patients who were anticipating leaving the hospital, many were concerned about not being ready to leave or being *‘thrown out’*. Concerns about the location, suitability and affordability of the next point of care were expressed. Younger and midlife patients tended to be concerned about the impact on their spouse/partner. A 55–year‐old male patient with multiple joint issues, wounds and infections expressed concern about the care providers’ suggestion to move into a long‐term care facility:

They [care providers] recommend that I go to a nursing home, which is just completely out of the question. I have a partner that has to be housed and I can't afford two locations.

(Transcript #75)

A 40‐year‐old male patient who was transferred to the hospital from another city was experiencing a downward spiral of health issues including kidney failure, two heart attacks, an infected bedsore and post‐surgical sciatic nerve damage. This was compounded by anticipated discontinuities in care, namely concerns that ‘*your people [providers at current hospital] are not familiar with what (name of city) offers’. (Transcript #22)*

Noted here is that supported transitions are needed as individuals enter and exit the hospital setting.

#### Bio‐psycho‐social care package

Patients talked about the need for social and mental health support, an optimal amount of physical rehabilitation, goal setting, and system navigation while in hospital. A 44‐year‐old female patient who was a widow and recently diagnosed with a rare neurological condition shared her experience.

I hear the same story from people from every walk of life. And that story is that no one is helping them with fear, with emotion, with frustration, with anger, with any type of emotional counselling. And I think that is as important or even more important in some cases than whether or not they get Humpty‐Dumpty‐ed in some sort of [physical/functional] way.

(Transcript #99)

To combat feelings of stress, patients commented that goal setting and expectation management should be a part of the care plan. A 53‐year‐old female patient who was suffering from diabetes and a brain tumour shared her thoughts:

Where am I supposed to be? Where is the projection? None of that is being given to me. And I'm confused as to where I am.

(Transcript #38)

Likewise, a 43‐year‐old female patient recovering from complications following surgery discussed the importance of communication around the care plan.

But I think if the person is coherent enough and wants to know, they should know. Or at least have somebody sit down and say, you know what, this is our plan for the next 2 weeks…

(Transcript #43)

Some suggested that the care plan should be managed by a system navigator; a person that would ‘take charge’ of the process. An 84‐year‐old female patient with both mental health and joint problems (osteoarthritis and osteoporosis) noted ‘*…when you've got multiple diseases like I have, it's to get one person that will sort of oversee everything. And there never is that person. That's what they need more of’. (Transcript #47)*

Finally, there was a strongly expressed need for more intensive physical rehabilitation, particularly among patients who were eager to re‐engage in their vocational and recreational activities. A 37‐year‐old male patient who had a work accident, which left him with multiple fractures and organ damage, articulated that it is important for providers to realize that many patients are ‘*[coming] from leading a very active life to doing nothing’. (Transcript #27)* To that end, this particular respondent along with many others wanted more opportunities to exercise, particularly on weekends when therapy was less available. Interest in exercising on their own, made possible through self‐management strategies, bed exercises taught by providers or access to the hospital gym after hours were expressed.

### Care capacity and quality

#### Optimal staff to patient ratio

When respondents spoke about their care experiences, they reflected on the skills and qualities of care providers that lend to better care including faster response times, better communication and consistency between providers and care units. They often simultaneously acknowledged the lack of capacity within the health‐care system, which they felt, precluded the ability of care providers to optimize their care experience.

Respondents noted the times when it was difficult to get staff attention; particularly during weekends where service provision would notably decrease, during ‘huddle times’ when providers would meet to discuss care plans, and shift changes. While waiting for responses was an expected characteristic of a busy care environment by some, others, particularly those with fluctuating and unpredictable health problems (i.e. irregular toileting patterns or those dependent on care providers to bring pain medication), were less tolerant and when accidents occurred felt *‘horribly embarrassed’*. A 58‐year‐old female patient with multiple health problems compounded by fractures shared her view on the consequence of provider capacity in the context of toileting.

They are understaffed here, I do know that. Like I understand understaffing and I think… I really think they are great nurses. I do. But because they have such a high patient load, generally I wish some of my needs had been met a bit faster. And the very basic one is toileting.

(Transcript #20)

#### Better provider communication

Patients identified that ‘*it's a matter of communication, not the abilities of the nurses’* and it was suggested that provider capacity be optimized by simply improving communication between staff. When patients used their call bells, it was not always clear when a health provider would respond or if they received the message at all. At times, communication issues also related to the language capacity of the care providers, demonstrating a need for staff ‘*to be educated on different languages’* with the ability to fully understand and comprehend the needs of the patients from a variety of ethnic backgrounds.

#### Consistency between providers

Patients commented that some providers went beyond the call of duty, despite notable constraints (e.g. lack of time), while others appeared less inclined to go the extra mile and felt that ‘*it's a lottery who you get’*. Lack of consistency was also noted among respondents who had transitioned from different care settings or within the hospital. A 58‐year‐old male patient who transitioned from a highly regimented therapy programme to transitional care comments that physical therapy ‘*has not been offered to me [on the new hospital unit] despite [asking] for it twice*’. *(Transcript #71)*

When lack of consistency was noted, some patients articulated what separated a good quality provider from a bad quality care provider.

A 56‐year‐old female patient with multiple sclerosis, osteoarthritis and a long history of joint problems differentiates high quality from low quality care:

A good listener. They actually listen to what you're saying. Non‐judgmental. They actually are there to help you. Whereas some of them, they're just here for the buck […] It's hard to describe it but there's no rapport, and a bit of a chip on the shoulder. That's about it.

(Transcript #55)

Likewise, a 51‐year‐old female patient who had arthritis and was recovering from her second total hip surgery and a post‐surgical medication interaction that placed her in a temporary coma shares her view:

…when they come in the room, they smile and they seem to be happy to do stuff. And if you ask them to do something that's not exactly, you know, the most glamorous part of nursing, that they are happy to do that for you. And you can just tell by them, that in their heart and soul, they were meant to be a nurse. They're not just here for pay day.

(Transcript #54)

The final theme speaks to the essence of the patient experience; the patient–provider relationship.

### The patient–provider relationship

#### Respect and dignity (know the patient)

Simply treating patients as valued persons emerged as a common theme. Many respondents discussed the importance of providers ‘*[not treating] us all the same’* and recognizing that [patients are] ‘*more than just a knee replacement’* and that the ‘*the patient is a person with all kinds of experience too’*.

An 82‐year‐old female patient with a hip fracture and arthritis talked about understanding the different capacities of patients:

Well, I think health care providers first of all could look at the person and get to know them and what they're capable of doing. Some are not, some are older, some are weaker. And they have to understand them. They can't just put them in a category of this is it. They're all different.

(Transcript #110)

A quality patient–provider relationship can be boiled down to *providing care* vs. *caring*. A 42‐year‐old female patient who had been recently diagnosed with a rare neurological illness shares this important contrast:

So professionally she follows up on any changes that are happening with me. But also professionally knowing that she's in a role that provides care and caring, which are 2 different things, she actively cares. She is caring. And I believe that that is the minimum standard of operation for somebody in health care.

(Transcript #99)

There were certain aspects of care that required greater attention to patient dignity, particularly bathing. A few female patients noted that it was critically important to have access to a provider of the same gender while bathing. A few respondents who had a negative experience in this regard detailed this part of their care as humiliating, and one woman expressed that she ‘*[would] rather die [than have a male nurse bathe her]’(Transcript #32)*

For other patients, attention to small non‐medical things made a huge difference in the care experience ‘*Like even getting water’ (Transcript #111)* or having access to a radio to listen to music. These simple things which can increase the quality of the care experience are often surmounted by the heavy medical care required by patients.

## Discussion

The themes generated in our analysis represent elements that could characterize a model of care for a highly complex patient population. The first theme (components of the care plan) speaks to ‘what needs to delivered’, while the second and third themes (care capacity and quality; patient–provider relationship) speak to ‘how the care should be delivered’.

The three broad themes were cross cutting – representing the views of patients across socio‐demographic variables (sex, age, education level, marital status, ethnicity, CCC/Rehab streams and perceived health severity). We did, however, note that certain subthemes were highly pertinent among certain populations. For example, issues related to dignity and bathing were only expressed by women. Also, the young–midlife population tended to be concerned about on‐going follow‐up on the care plan (i.e. goal setting) and were concerned about the impact on their loved ones (spouses and parents) following a transition back home. We suspect that given the life stage of the young–midlife population (i.e. in the midst of careers and childrearing), it was critically important to transition back to their lives and routines.

### Theme 1–components of the care plan

The quality of the care experience starts with a comprehensive assessment so that important aspects of the patient including their physical functioning, illness history and mental health status can be captured. Further, synchronizing data collection to reduce redundancy and minimize burden on patients is needed, while drawing on family caregivers’ knowledge can help piece together the history and needs of the patient.

Data systems that are currently in place at the hospital of study and across health settings inadequately capture the full range of needs of complex patients.^23^ There are currently robust measures used in hospitals, long‐term care facilities and primary care settings that emphasize biomedical factors (disease types, counts and severity) as well as utilization (treatment time, hospitalization), creating a vital picture for some aspects of complexity. These elements are critical, but what is required is the integration of psychosocial factors and patient experience, aspects that tend to be underrepresented in current measures.^24^, ^25^

In addition to a proper assessment, supported transitions for patients are needed as they move into and out of care settings. People who have multiple, on‐going care issues tend to require care from multiple providers and organizations across the care continuum, yet each health‐care setting tends to operate as a disparate entity making care navigation and mobilization of resources challenging.^26^ Poor transitions may place patients in a vulnerable state which can set the stage for a negative care experience. What is needed in the care context is greater attention to care coordination across settings and geographical boundaries; linkages to peer patients (previously hospitalized patients who ‘know the ropes’); and moral support from care providers regarding the management of expectations during transitions.

In addition to transitions, the components of the care package should match the needs of patients. There is a growing body of literature demonstrating that chronic disease constitutes more than the sum and severity of physical health problems. Chronic disease has been linked to depression,^27^ psychological distress,^7^ social exclusion, poverty^28^ and relationship strain.^6^ For complex patients, intensive medical, social and self‐management support^29^, ^30^ is required and can potentially improve outcomes.^31^ Importantly, incorporating health and social care into care plans has been linked to more efficient use of resources.^24^, ^32^ The type of care provision desired by the respondents in our study complement these findings. Mental health support, coaching and goal setting, access to more active physical therapy; system navigation; and support for ‘the small things’ which typically fell outside the realm of medical needs (i.e. someone to bring water to the bedside) were expressed needs among patients with CCD.

### Theme 2–care capacity and quality

Current health systems, with their acute, episodic orientation, are ill‐fitted to meet the needs of modern patients.^33^ Movement towards team‐based interdisciplinary care is increasing yet providers continue to train in professional silos and are given limited capacity to work to their full scopes of practice in the workplace.^34^ Institutional structures (i.e. fee schedules that are tied to care episodes, volume constraints and efficiency agendas) limit the extent to which care can be patient centred and comprehensive. For individuals who require only acute intervention or surgery, the system as it stands is (more or less) equipped. However, when individuals are characterized by multiple co‐occurring and fluctuating health problems, the current model of health care becomes problematic. Although evidence on the benefits of alternative models of care (i.e. integrated care models) for individuals with complex health problems exist,^31^ this is mostly at the community and primary care levels, and the scalability of such interventions have been minimal due, in part, to the institutional constraints noted above.

Interestingly, even in a system of care that allows little time for providers to embed a holistic approach, it appeared that some providers in our study were able to do so. The patients in our study noted differences in the quality of care from provider to provider, as well as differences between care settings and hospital units. Providing consistent quality care in a care environment that is stretched to capacity is challenging, but requires attention in order to optimize the patient experience. Further research is required to understand these nuances, including how they can be addressed at an organizational level.

### Theme 3–the patient–provider relationship

Patients talked about the importance of on‐going communication, attention to personal needs and learning styles and being acknowledged and validated in the care process. Differences between basic and holistic care emerged as patients commented on the care providers who went the extra mile and how this enhanced their overall care experience.

Literature on patient centredness is not new in clinical medicine; dating back to the 1950s with Carl Rogers's concept of client‐centred therapy 35 and further in the 1980s with Stewart's model of patient‐centred care.^36^, ^37^ Our findings reinforce the components of Stewart's model particularly the components that speak to understanding the illness experience; incorporating the whole person in care planning; finding common ground; enhancing the patient–care provider relationship and being realistic. Our findings also coincide with research conducted by Kitson *et al*.^38^ who conducted a narrative synthesis on the core elements of patient‐centred care which elucidated three core themes: the importance of patient participation, patient–provider relationships and the context in which care is delivered.

In addition to research on patient‐centred care, these findings complement the existing literature on the specific components of care delivery that are needed, particularly for individuals with CCD including the importance of personalized, self‐management support,^18^ clearly communicated and individualized care plans,^13^ system navigation and coordination,^13^, ^16^ continuity of care and seamless discharges.^16^ What our study adds is greater insight into the complex care environments in which providers work; the characteristics and inconsistencies in care provision; the necessary components of care packages (inclusive of mental and social health support); and the granular aspects of care that are important to patients (communication, dignity, personalized, tailored care) that are required for complex patients who spend a fair amount of time in the health‐care system. What is less clear in the existing literature is how to operationalize these patient‐centred components into practice.^38^ Our findings reinforce the importance of being patient centred in practice, but further work is needed to determine how to tailor and embed these pieces into interventions for complex patients in complex care environments.

## Conclusion

The key challenge going forward is meeting the needs of individuals with CCD within an environment constrained by lack of resources (i.e. human resources and capital) and funding structures that incent cure over care. Care settings that serve individuals with CCD require staff that are given the flexibility in their role to adapt care to the personal needs of patients—which include physical, social and mental health support and the capacity to respond to fluctuating health problems. It is essential for providers to have the tools (i.e. training and capacity) to assist patients in coming to terms with their care regimens, life changes and next points of care. This requires highly trained clinical staff intermixed with providers and volunteers who can address instrumental needs such as social support. Triaging patients into complex and less complex categories, with corresponding ratios of providers, may create the opportunity to better address varying levels of complexity in health services delivery.^39^

As health systems throughout the industrialized world move to sustain health budgets while optimizing quality of care, it is critical to better understand this population, so that appropriate metrics, services and policies can be developed. The study has generated a body of evidence on the important components of care delivery from the perspectives of a diverse group of chronically ill individuals who have spent a considerable amount of time in the health‐care system. Moving forward, exploration around the appropriate funding models and skill mix is needed to move the evidence into changed practice.

## Limitations

The study provides insight on the care delivery experience from one hospital‐based population with CCD. The use of a single site convenience sample limits the transferability of the study findings. Further research is required to validate the needs of highly complex patients in other complex continuing care and rehabilitation settings as well as across the continuum of care (e.g. homecare, primary care, long‐term care, acute care). Although the themes represented in our findings represented patients across socio‐demographics, disease types and severities, a more nuanced and in‐depth analysis is needed in future papers, so specific typologies of complex patients (i.e. those who are young‐midlife) can be better understood and interventions appropriately tailored.

## Sources of funding

The corresponding author received salary support from the Great‐West Life, London Life, Canada Life New Scientist Fund for the program of work within which this study was embedded. The funding for the study came predominantly from Bridgepoint Health in Toronto, Ontario, Canada.

## Author's contributions

KK contributed to the study design, data analysis and the design of the manuscript along with drafts and revisions. SH contributed to data collection, data analysis and manuscript revisions. AS participated in the study design and revisions of the manuscript. CA participated in the study design and revisions of the manuscript. RL participated in the study design and revisions of the manuscript. RT conducted a scan of the literature for the background section of the manuscript and revisions of the manuscript. CB contributed to data collection and revisions of the manuscript.

## Conflicts of interest

The authors declare no conflicts of interest.

## Ethics approval

Ethical approval was received on January 26, 2011 by the Joint Bridgepoint‐West Park‐Toronto Central Community Care Access Centre Research Ethics Board.
